# Advances in the Use of Microwaves for Isolation and Structural Modification of Biobased Polysaccharides

**DOI:** 10.1021/acsomega.5c11982

**Published:** 2026-03-04

**Authors:** Euda Maria Gomes dos Santos, Expedito Lopes Fernandes Júnior, Gabrielle de Lima Maniçoba, Amanda Damasceno Leão, Antônia Carla de Jesus Oliveira, Luíse Lopes Chaves, Monica Felts De La Roca Soares, José Lamartine Soares-Sobrinho

**Affiliations:** Quality Control Core of Medicines and Correlates − NCQMC, Department of Pharmaceutical Sciences, 28116 Federal University of Pernambuco, Recife, Pernambuco 50670-901, Brazil

## Abstract

Microwave technology has become a prominent and sustainable method for isolating and chemically modifying natural polysaccharides, mainly from plant biomass. It offers rapid, uniform, and selective heating, significantly reducing reaction times and energy use compared with traditional methods. During isolation, microwaves enable the effective extraction of high-purity polysaccharides using smaller amounts of milder solvents, aligning with green chemistry principles. For chemical modifications, microwave activation enhances reaction efficiency, lowers reagent consumption, and minimizes byproduct formation. These improvements produce polysaccharides with enhanced properties, such as increased solubility, antimicrobial activity, and tailored functionalities, broadening their use in food, cosmetics, pharmaceuticals, and biodegradable packaging industries. Compared with conventional heating, microwave-assisted processes have been reported to reduce energy demand and the associated carbon footprint. Future developments include integrating microwaves with other activation techniques such as ultrasound or enzymatic catalysis to boost efficiency and selectivity. Utilizing residual biomass as a polysaccharide source also presents a promising route toward sustainable and circular economy practices, meeting rising demands for eco-friendly materials and clean technologies. Overall, microwave-assisted processes are establishing themselves as vital tools in green chemistry, driving innovation in renewable materials and sustainable industrial applications.

## Graphical abstract

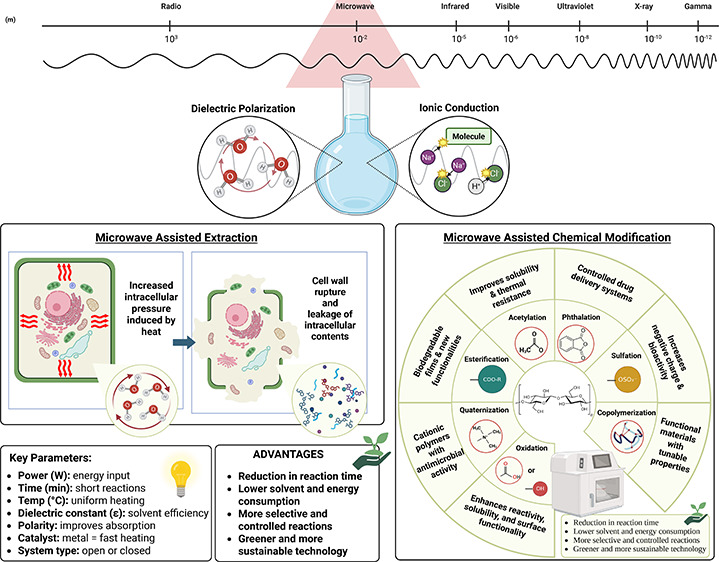

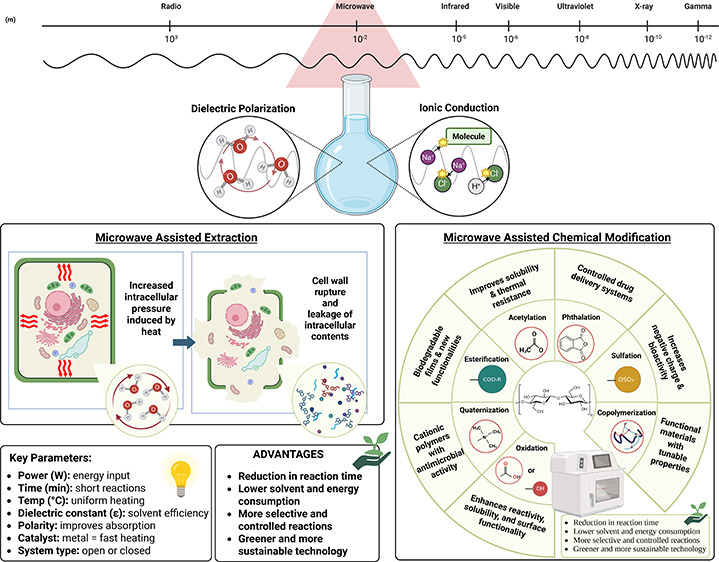

## 1 Introduction

Natural polysaccharides are of great biological, industrial, and environmental importance due to their functional versatility and wide availability in renewable sources. Biologically, they play essential roles as structural components, such as cellulose in cell walls, and as energy reservoirs, such as starch in seeds and tubers. Due to their chemical and functional versatility, biodegradability, and biocompatibility, their application in various areas has been explored.^1^

Industrially, they are used in various areas: in food, as thickeners and stabilizers; in pharmaceuticals, as excipients and bioactive agents; and in the production of sustainable materials, such as bioplastics and biodegradable packaging.^2^ In addition, natural polysaccharides have great potential for innovative applications, such as tissue engineering, due to their biocompatibility and chemical modification capacity.^3^ Their abundance and biodegradability also make them crucial for initiatives aimed at the circular economy and reducing dependence on fossil fuel-derived materials.^4^

In the food industry, they play an important role as thickeners, stabilizers, and gel formers in foods, such as sauces, yogurts, and desserts. Their nutritional properties have also been explored in the production of functional foods with higher nutritional value.^5^ In the pharmaceutical industry, polysaccharides are prominent in the production of controlled drug delivery systems,^6^ pharmaceutical excipients,^7^ production of hydrogels and biomaterials, in addition to their own use as bioactive agents.^8^

Polysaccharides are relevant across multiple disciplines and contribute to advances in science, technology, and sustainability. Their growing use reflects not only the search for innovative solutions but also the need for more environmentally friendly and accessible processes. In this perspective, traditional methods of isolation and chemical modification of polysaccharides face technical, environmental, and economic challenges that limit their efficiency and applicability.^9,10^

In isolation, conventional techniques often require long processing times, high temperatures, and the intensive use of chemical solvents, such as acids or alkalis, which can degrade the structure of polysaccharides and reduce their functionality.^11^ In addition, these processes generate chemical waste that increases treatment costs and negatively impacts the environment.^12^

In chemical modification, challenges include low reaction selectivity, which can generate unwanted byproducts, hindering purification and compromising yield.^13^ Another obstacle is the need for harsh conditions, such as high pressure or the use of toxic catalysts, which increase operating costs and limit the sustainability of the processes. These problems also hinder scalability for industrial applications.^14^

There is a growing need for more sustainable and efficient approaches, such as the use of emerging technologies, including microwave heating, ultrasound, and enzymatic processes, which promise to overcome these limitations, reducing environmental impact and increasing economic viability.^15^ The use of microwaves is noteworthy in this regard, as it is a fast and efficient method of heating, enabling a reduction in the use of solvents and precise control of temperature and pressure conditions, optimizing the extraction and modification of polysaccharides.^16^

In isolation, microwaves can accelerate the extraction of polysaccharides from different sources, allowing for higher yields with reduced material degradation.^17^ This approach is also considered more environmentally friendly, as it can reduce the use of harsh chemicals and organic solvents commonly employed in traditional extraction methods.^18,19^ However, beyond these general advantages, the relevance of microwave-assisted extraction becomes particularly evident when viewed in the context of a fundamental technical limitation that persists across polysaccharide extraction methodologies.

One of the major technical bottlenecks in the isolation of polysaccharides from natural matrices lies in the inherent trade-off between achieving high extraction yields and preserving the structural integrity of these macromolecules, particularly with respect to molecular weight retention and associated bioactivity. Conventional extraction processes based on conductive heating often require prolonged processing times, leading to high cumulative thermal exposure and favoring depolymerization and structural degradation. In this context, microwave-assisted extraction emerges as a promising solution to this long-standing challenge, as it enables rapid and more homogeneous volumetric heating, thereby significantly reducing the overall processing time and cumulative thermal load. As a result, when microwave power and irradiation time are carefully controlled within appropriate operational windows, MAE allows for high extraction yields to be achieved while minimizing structural degradation.

In addition, the use of microwaves for chemical modifications allows for more selective reactions in less time, resulting in products with desirable characteristics and greater control over the final properties.^20^ This approach may also be more sustainable, as it offers an alternative to conventional processes that consume high amounts of energy and large volumes of solvents, aligning with trends toward reducing environmental impacts in industry.^21^ With the advancement of research and the development of more accessible equipment, the use of microwaves is an effective and efficient solution for industry, promoting greener and more economical production of modified polysaccharides.

Beyond providing a comprehensive survey of microwave-assisted methodologies for the isolation and chemical modification of biobased polysaccharides, this review introduces an integrative conceptual framework that links the dielectric properties of the system (dielectric constant, dielectric loss factor, and tan δ), operational parameters (microwave power, irradiation time, temperature, and solvent selection), and the resulting structural and functional outcomes of the extracted or modified polysaccharides (yield, preservation, degree of substitution, and bioactivity). By explicitly connecting the physical mechanisms of microwave–matter interactions with experimentally reported performance metrics, this review shifts the discussion from predominantly empirical optimization toward a physicochemical rationalization of microwave-assisted processes. This framework enables a clearer interpretation of discrepancies across studies, highlights operational boundaries, and provides more robust guidelines for process design, optimization, and industrial scalability of microwave-assisted technologies applied to biobased polysaccharides.

## 2 Principles of Microwave Use in Chemistry

Microwaves are nonionizing electromagnetic radiation located between radio waves and infrared radiation, which propagate through the simultaneous and mutually sustained oscillation of perpendicular electric and magnetic fields, allowing energy to be transferred through space. This oscillation enables efficient interaction with matter, and because they do not have enough energy to ionize atoms or break chemical bonds, they differ from ionizing radiation such as X-rays and γ-rays, making them safe for various chemical applications. In chemistry, this radiation is widely used to promote internal and volumetric heating of materials, based on the interaction of radiation with molecular dipoles and ions present in substances, providing faster, more selective, and more efficient reactions compared to conventional thermal methods.^22^

From a technical standpoint, microwave equipment used in chemical synthesis and processing is considered safe and offers notable advantages over traditional thermal heating methods. Among the most relevant benefits are a significant reduction in processing time, energy savings, greater precision in controlling process parameters, and rapid activation and interruption of irradiation, which gives the system high reproducibility and efficiency.^23,24^ These advantages make microwaves a strategic tool in green chemistry, aligning with the principles of sustainability and innovation in synthetic processes.

The heating of materials by microwaves occurs through the direct conversion of electromagnetic energy into thermal energy, a process that occurs predominantly through two physical mechanisms: dipole rotation and ionic conduction, as illustrated in Figure 1. Dipole rotation is related to the interaction of the oscillating electric field with polar molecules, i.e., those that have a permanent dipole moment.^25^ The constant reorientation of these molecules in response to the alternating field results in intermolecular friction, generating heat efficiently and locally. In turn, ionic conduction occurs when charged species, such as ions present in the sample, are mobilized by an electric field. This oscillatory movement generates collisions and, consequently, energy dissipation in the form of heat. In heterogeneous materials or materials with significant surface charge, interfacial polarization can arise as a combination of these mechanisms, amplifying heating through the creation of localized potential gradients.^26^

**Figure 1:**
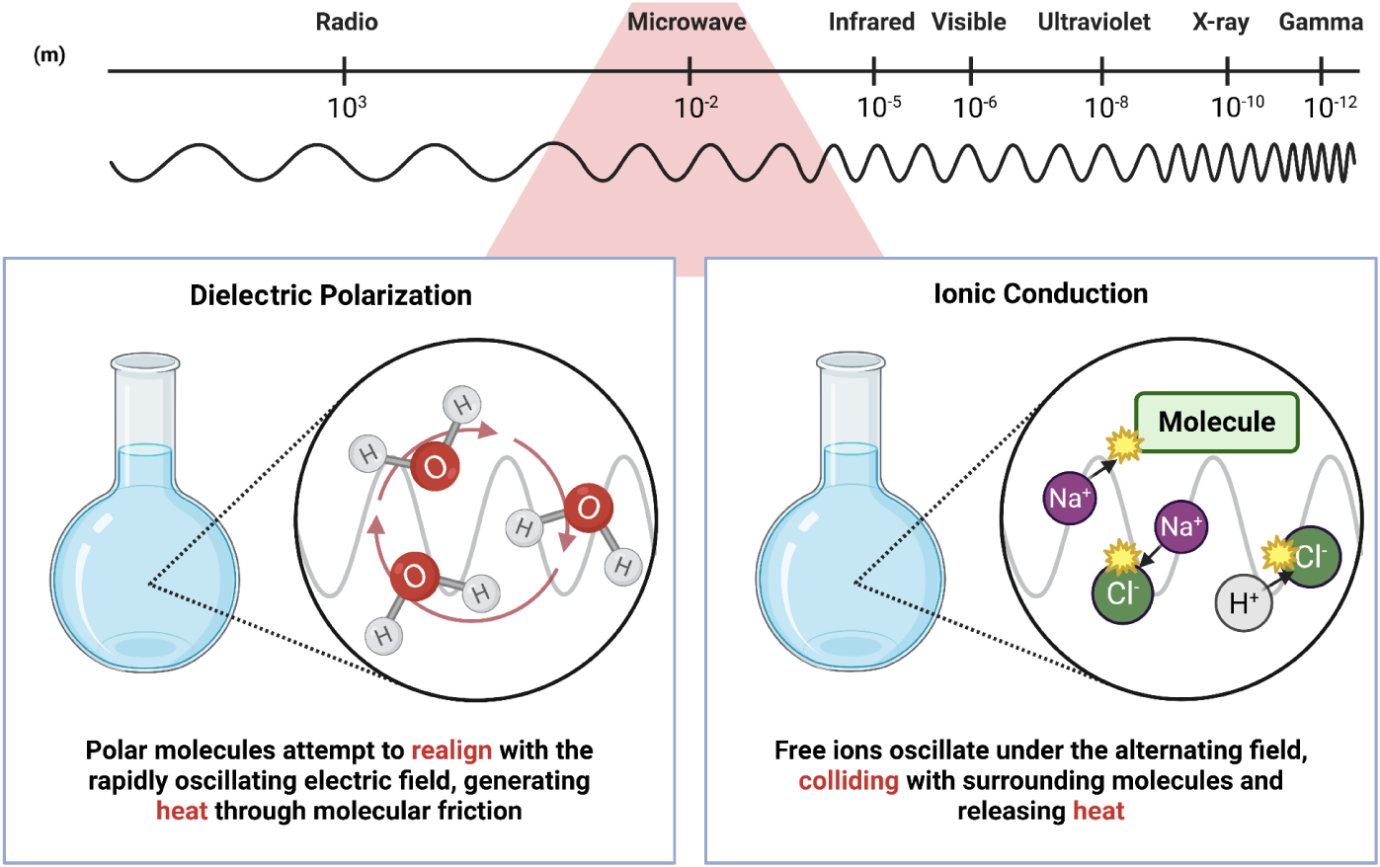
Microwave heating mechanisms.

The efficiency of this heating process is intrinsically related to the dielectric properties of the materials, which determine their ability to interact with microwaves. The dielectric constant (ε′) represents the material’s ability to polarize when subjected to an electric field, serving as an indicator of the amount of energy that can be absorbed. The dielectric loss factor (ε″), in turn, is associated with the efficiency with which this absorbed energy is converted into heat. The ratio between these two parameters, known as the dissipation factor (tan δ), is used to estimate the susceptibility of a given material to microwave heating. High tan δ values indicate that the material effectively absorbs radiation and converts it into thermal energy efficiently.^27^ In addition to these properties, physical factors such as viscosity, density, and particle size also have a significant influence on heating, as they modulate molecular mobility and the way heat is distributed in the medium.^28,29^

In terms of application, the scale of operation directly influences the configuration of the microwave systems. Single-mode equipment, generally used in laboratory research, is designed for small volumes—typically up to 200 mL—and operates at powers between 100 and 300 W. In these systems, the cavity is designed to concentrate radiation on the sample, optimizing energy efficiency and heating uniformity.^30^ On the other hand, larger multimode equipment is used for industrial or pilot-scale processes, requiring higher power due to the dispersion of waves over a larger area. In these cases, controlling radiation distribution and thermal homogeneity becomes more complex, requiring complementary strategies such as sample rotation or the use of auxiliary dielectric materials.^31^

A rapidly expanding field for the use of microwaves is the extraction of bioactive compounds from natural matrices. The technique, recognized as a promising alternative to conventional approaches, combines selectivity, speed, and reduced solvent use, making it a sustainable and effective method. The efficiency of this technique is related to the ability of microwaves to penetrate the cellular structure, promoting internal heating of the cells due to the absorption of radiation by water and other polar constituents present in the matrix. The increase in temperature generates sufficient internal pressure to break the cell membranes, facilitating the release of intracellular content, such as essential oils, flavonoids, alkaloids, and other secondary metabolites of pharmaceutical, cosmetic, and food interest.^32–34^ This process, also known as “microwave-assisted thermal rupture,” has been extensively studied and can be visually understood through schematic representations, such as the one in Figure 2, which illustrates the sequence of cellular events during extraction from plant cells.^29,35^

**Figure 2:**
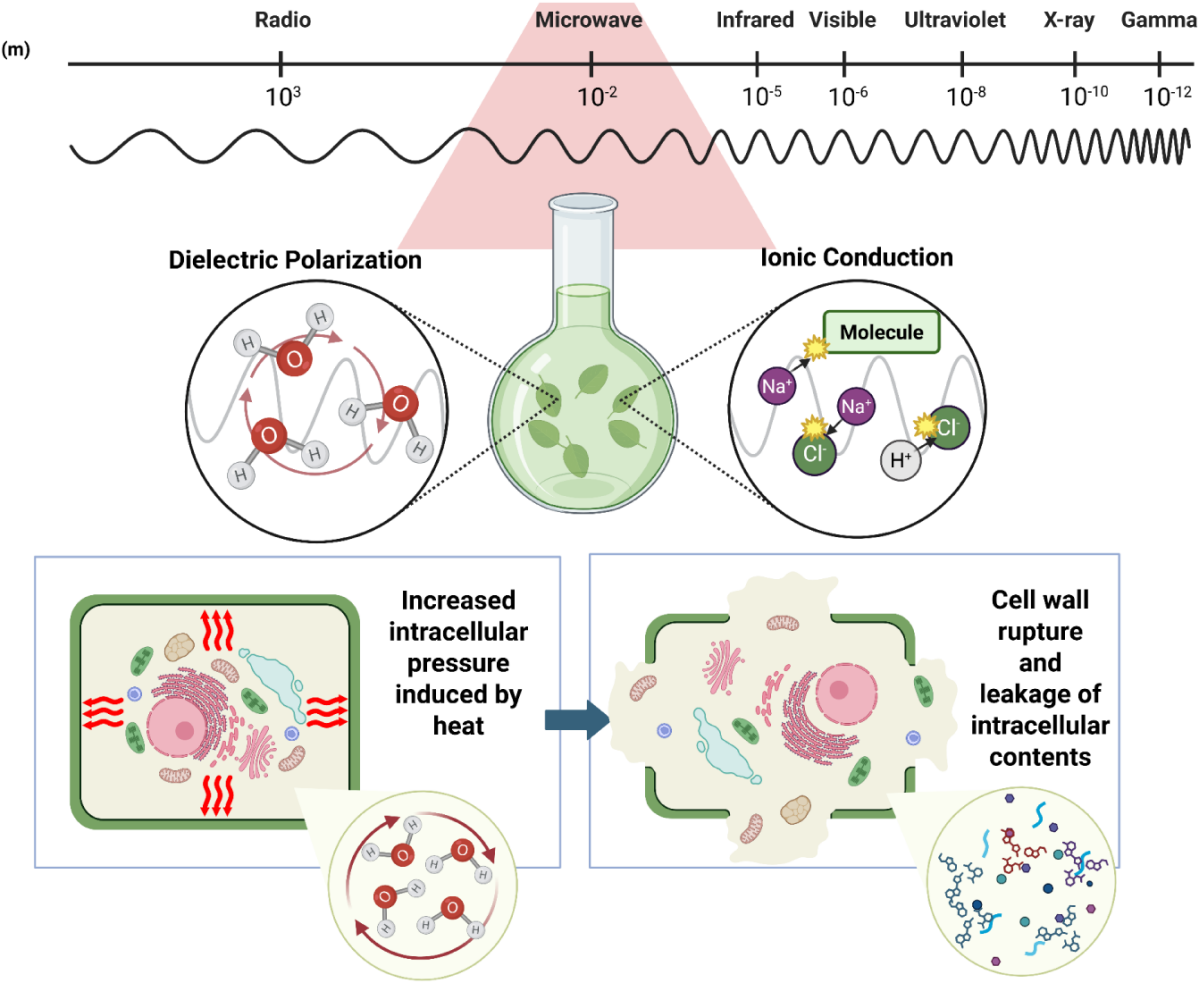
Microwave-assisted extraction in plant cells.

Thus, the principles of microwave use in chemistry not only expand the technical possibilities for synthesis, extraction, and modification of materials but also promote a more sustainable and efficient paradigm for the development of chemical processes. A detailed understanding of the physical mechanisms, the dielectric properties of materials, and the suitability of the scale of operation is fundamental to the full exploitation of this technology in a contemporary scientific and industrial context.

## 3 Parameters That Influence Microwave-Assisted Extraction

Microwave-assisted extraction was established as an efficient and sustainable technique for obtaining bioactive compounds from biobased matrices. It is influenced by several operational parameters that directly impact its yield, selectivity, and quality of the extract obtained. Among these, extraction time, temperature, applied power, and type of solvent used stand out, all of which must be carefully adjusted to optimize the process.

### 3.1 Time

Extraction time is a key variable that determines the extent and efficiency of the compound transfer from the matrix to the solvent. One of the main advantages of microwave extraction is the speed with which heating occurs, allowing processes that traditionally take hours to be carried out in minutes.^36^ However, this speed requires fine timing, since insufficient extraction periods can result in reduced yield due to the absence of sufficient thermal energy to promote adequate cell wall rupture and metabolite release.

On the other hand, excessively long times can be harmful, since prolonged exposure to heat can induce thermal degradation of target compounds, especially those that are heat-sensitive, such as polysaccharides, flavonoids, and antioxidants, compromising the quality of the final extract. In addition, long exposures can promote secondary reactions, altering the chemical composition and biological activity of the extracts.^37,38^ Therefore, determining the optimal extraction time is crucial to balance the efficiency and preservation of bioactive compounds.

The extraction times observed in the studies described in Table 1 ranged from 6 to 20 min, directly reflecting the critical role of this variable in balancing process efficiency and the integrity of the extracted compounds. Evidence suggests that, under adequate power conditions, short times—between 6 and 7 min—are sufficient to promote high-yield extractions. This is the case in the studies by Zhao et al.^19^ and Su et al.,^39^ in which the rapid interaction between microwave radiation and the solvent promoted the release of the target compounds without the need for prolonged exposure. In these contexts, the combination of high power and appropriate solvents favors the diffusion of compounds in a short time, avoiding losses due to prolonged thermal degradation.

**Table 1:** Optimal Conditions for Polysaccharide Extraction from Different Plant Matrices

Species	Matrix	Power	Solvent	Time	Temperature	Yield (%)	Configuration	References
*Althaea officinalis* L.	Roots	430 W	Water|Proportion 40:1 (mL/g)	20 min	60 °C	14.51 ± 0.06%	Home microwave oven	^41^
*Chuanminshen violaceum*	Roots	466 W	Water|Proportion 40:1 (mL/g)	15 min	64.5 °C	34.59 ± 0.51%	Home microwave oven	^42^
*Citrus unshiu*	Fruit peel	400 W	Acid solution (HCl)|3 g in 500 mL	7 min	-	28.0 ± 0.5%	Home microwave oven	^39^
*Panax ginseng*	Roots	550 W	Water|Proportion 30:1 (mL/g)	6 min	70 °C	41.6% ± 0.09%	Multimode microwave	^19^
*Hippophae rhamnoides L*.	Fruits	600 W	Water|Proportion 10:1 (mL/g)	6 min	85 °C	0.264% ± 0.005%	Multimode microwave	^43^
*Morus nigra*	Leaves	170 W	Water|Proportion 28.2:1 (mL/g)	10 min	-	9.41%	Multimode microwave	^37^
*Porphyra haitanensis*	Whole plant	77.84 W	Water|Proportion 28.98:1 (mL/g)	14.14 min	-	5.01 ± 0.32%	Multimode microwave	^40^
*Auricularia polytricha*	Fruits	59 W	Water|Proportion 22:1 (mL/g)	15.3 min	-	4.10%	Multimode microwave	^44^
*Camptotheca acuminata*	Fruits	570 W	Water|Proportion 30:1 (mL/g)	20 min	-	6.81 ± 0.04%	Multimode microwave	^45^

However, this relationship is neither linear nor universal. As shown by Chen and Xue,^40^ longer times are necessary when using lower powers. This is due to the lower energy supply per unit of time, which requires extended exposure to ensure cell breakage and complete diffusion of the metabolites from the matrix. The longer duration compensates for the gentler heating, which is especially advantageous when the goal is to preserve heat-sensitive compounds

Additionally, the study by Wei et al.^43^ makes a crucial observation: even with short exposure times, the yield can be extremely low (0.264%), suggesting that, in addition to power, the nature of the matrix and the concentration of the compounds influence the effectiveness of the chosen exposure time. In such cases, insufficient time may not allow the radiation to adequately penetrate the layers of the sample or allow sufficient time for the diffusion and solvation of the target compounds.

In conclusion, extraction time must be carefully optimized in conjunction with other parameters—especially power, matrix type, and solvent—because process efficiency depends not only on absolute time but also on the synergy between operational variables. Short times may be desirable from an energy and productivity standpoint but only when supported by conditions that ensure effective and selective extraction.

### 3.2 Power

The power of microwave equipment, determined by the energy emitted by the magnetron, is a critical parameter that directly influences the heating rate and, consequently, the time required for extraction. This variable is directly related to the amount of electromagnetic energy supplied to the system per unit of time, and its conversion into thermal energy affects the speed at which compounds are released from the matrix into the solvent.^46,47^

Higher powers provide a rapid increase in sample temperature, reducing the total extraction time, as shown by Zhao et al.^19^ and Sun et al.^45^ However, this intense energy supply can cause uneven heating, physical damage to cell walls, and thermal or chemical degradation of compounds, especially if it exceeds the thermal capacity of the sample or is applied for prolonged periods. These conditions can result in the loss of volatile or heat-sensitive compounds, compromising the quality of the extract.

On the other hand, very low powers, such as those used by Chen and Xue,^40^ promote slower extractions with lower yields. Although these conditions are suitable for compounds that are more sensitive to heat, their low energy efficiency requires long irradiation times, which may be impractical in production contexts and may also favor unwanted secondary reactions.

Studies such as those done by Hashemifesharaki et al.^41^ and Dong et al.^42^ show that moderate powers, between 400 and 500 W, offer a good balance between extraction efficiency and preservation of the chemical integrity of the compounds. This power range provides a controlled and effective heating rate, sufficient to promote the breakdown of cell structures without inducing thermal degradation of the most fragile constituents. In such cases, the literature recommends combining intermediate powers with longer exposure times to promote gradual and selective extraction.^36^

In addition, the ideal power should consider the type of cavity of the equipment used. In microwave systems with single-mode cavities, where energy is directed in a more concentrated and uniform manner, lower powers may be sufficient to achieve high effective temperatures. In multimode cavities, which operate with larger volumes and less homogeneous energy distribution, higher power levels are required to ensure uniform and efficient heating throughout the sample.^48^

The influence of microwave power and irradiation time on the extraction performance and polysaccharide integrity reported in the literature is summarized in Table 2. Therefore, the choice of power must be made in an integrated manner with the other parameters of the process, such as time, temperature, type of solvent, and characteristics of the matrix. Careful adjustment of this parameter is essential not only to maximize extraction yield but also to ensure the quality, selectivity, and sustainability of the process.

**Table 2:** Influence of Microwave Power and Irradiation Time on Extraction Efficiency and Structural Integrity of Polysaccharides

**Microwave power range**	Low (50–150 W)	Medium (300–500 W)	High (≥600 W)
**Irradiation time**	10–30 min	5–20 min	1–10 min
**Typical outcomes**	Moderate yields, high molecular weight preservation	High yields with acceptable structural integrity	Very fast extraction, high initial yields
**Main advantages**	Mild conditions, low degradation	Optimal balance between efficiency and preservation	Maximum productivity, short processing time
**Potential risks**	Lower extraction efficiency	Localized overheating if poorly controlled	Depolymerization, loss of bioactivity
**Suitable applications**	Thermolabile polysaccharides (e.g., β-glucans, sulfated polysaccharides)	Seeds, peels, and moderately dense plant matrices	Dense or highly hydrated matrices when short exposure is ensured

### 3.3 Solvents

The choice of solvent in microwave-assisted extraction is one of the most decisive factors for the efficiency, selectivity, and sustainability of the process. This variable directly influences radiation absorption, the solubilization of target compounds, and the environmental impact of extraction. Solvents with a high dielectric constant (ε′) and high dissipation factor (ε″) absorb microwave energy more efficiently, converting it into internal heat quickly and evenly. This volumetric heating promotes the breakdown of cell structures and the release of analytes, optimizing the kinetics of the process.^49^

Water is the most widely used solvent, as evidenced by several studies,^19,41,42^ due to its high dielectric constant, high polarity, and excellent compatibility with water-soluble compounds. In addition, its availability, low cost, and lack of toxicity make it highly attractive for applications that follow the principles of green chemistry. Water promotes efficient heating, even at low irradiation times, and facilitates rapid and highly selective extractions.^29^

However, water is not universally effective, especially for low-polarity compounds or structures that are more resistant to hydrolysis. In such cases, alternative solvents, such as acidic, alcoholic, or ionic solutions, can be applied strategically. For example, Su et al.^39^ used an HCl solution for pectin extraction, demonstrating that the pH of the medium directly influences the breakdown of interactions between polysaccharides and the matrix, significantly increasing yield. This shows that in addition to interaction with microwaves, the chemical affinity between solvent, matrix, and analyte is equally decisive.

Polar solvents and ionic solutions, because of their permanent dipole moments, also interact strongly with radiation, promoting rapid and controlled temperature increases and, thus, more efficient extractions.^50^ Nonpolar solvents, on the other hand, due to their low dielectric constant, tend to absorb a small amount of microwave energy, resulting in slow heating and less effective extractions. In such cases, mixed solvents or cosolvents can be used to broaden the spectrum of extracted compounds and balance the thermal efficiency and chemical selectivity of the process.

Another key aspect is the solvent/matrix ratio, which influences the yield and dynamics of the process. Higher ratios favor gradual and more controlled extraction, as observed by Chen and Xue,^40^ as they increase the availability of the extraction medium and facilitate heat dissipation. In contrast, very low ratios can limit the solubilization of compounds and favor uneven heating, compromising both the yield and stability of the extracts, as demonstrated by Wei et al.^43^

Therefore, the choice of the ideal solvent must take into account multiple factors: dielectric properties, polarity, toxicity, affinity with target compounds, sustainability, and proportion in relation to the matrix. The right combination of solvent, time, power, and temperature not only maximizes the yield but also ensures the quality of the extract and the alignment of the process with environmentally responsible practices.

### 3.4 Temperature

Temperature is one of the most critical parameters in microwave-assisted extraction, as it results from the direct conversion of electromagnetic energy into thermal energy inside the sample. This internal and volumetric heating promotes the softening and destruction of plant tissues, facilitating the diffusion of compounds into the solvent. In addition, the increase in temperature provides sufficient energy to break intermolecular bonds present in the solid matrix, favoring the solubilization and release of polysaccharides and other bioactive metabolites.^51^ As a result, the extraction kinetics are accelerated, significantly reducing the time required to obtain high-yield extracts.

Moderate temperatures, generally between 60 and 70 °C, have proven effective in promoting high-yield extractions while maintaining the integrity of the extracted compounds. Studies such as those by Hashemifesharaki et al.,^41^ Dong et al.,^42^ and Zhao et al.^19^ reinforce that this temperature range is sufficient to optimize process efficiency without inducing significant degradation. However, excessive temperature increases can compromise the quality of the extract, especially in systems such as microwaves, where heating is rapid, heterogeneous, and difficult to control in multimode cavities.

When the temperature exceeds the stability limits of the compounds, as in the case of 85 °C reported by Wei et al.,^43^ not only there is a reduction in yield but also thermal degradation of sensitive compounds, in addition to possible unwanted reactions, such as oxidation or the loss of volatile compounds. These changes directly impact the selectivity of the process, the final chemical composition, and the functional value of the extract. Therefore, strict and continuous temperature control throughout the microwave-assisted extraction process is essential. This monitoring ensures not only the effectiveness of the extraction but also the preservation of the quality of the target compounds. Thus, temperature should not be treated merely as an operational variable but as a determining factor in the performance, safety, and applicability of the extract obtained.

To facilitate the rational selection and optimization of microwave-assisted extraction parameters, Figure 3 presents a decision flowchart that guides the sequential adjustment of key variables involved in MAE processes. The proposed scheme integrates fundamental characteristics of the biomass matrix, thermal sensitivity of the target polysaccharide, solvent dielectric properties, and operational microwave conditions. By simultaneously considering the extraction yield and molecular weight preservation as critical performance criteria, this flowchart provides a practical and transferable framework to support process design, reduce empirical trial-and-error approaches, and improve reproducibility across different polysaccharide extraction systems.

**Figure 3:**
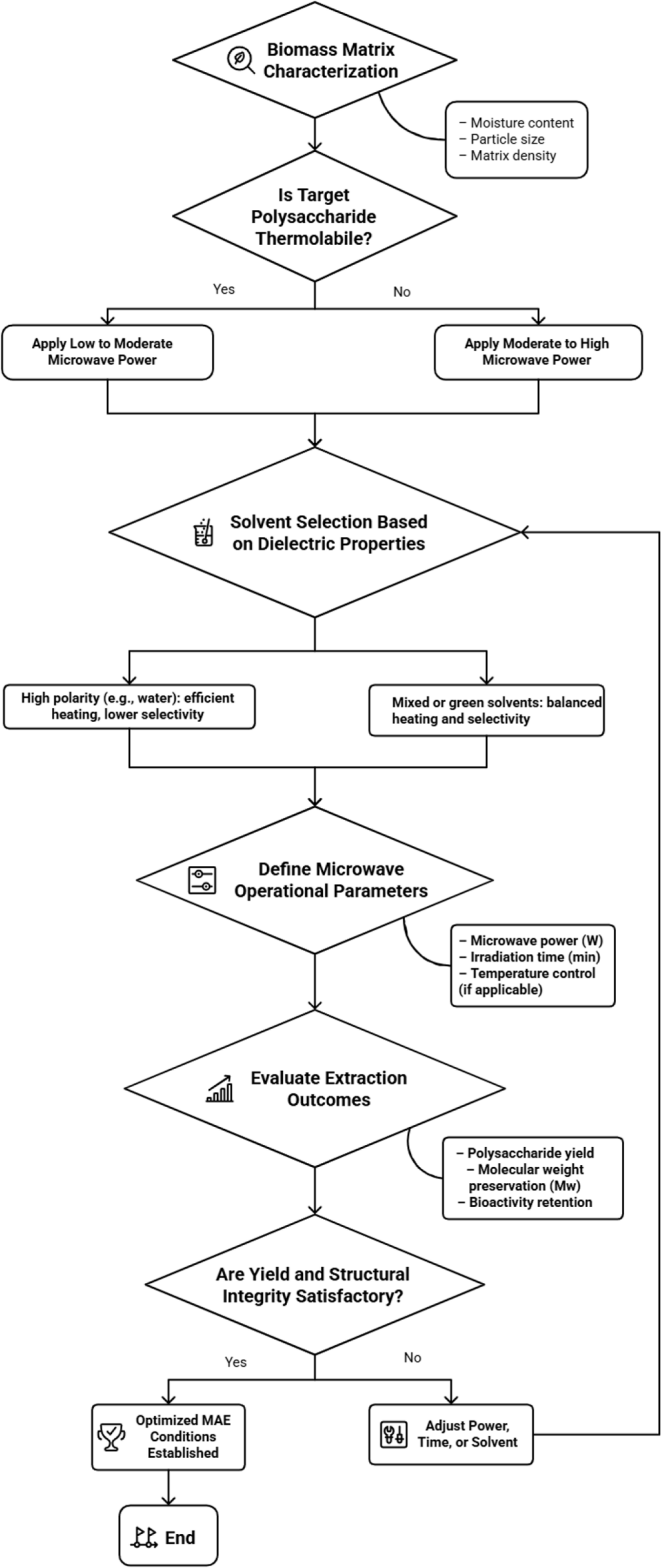
Decision flowchart for the optimization of microwave-assisted extraction (MAE) parameters, based on biomass matrix characteristics, solvent dielectric properties, and operational microwave conditions, guided by extraction yield and molecular weight preservation. Schematic illustration generated with the assistance of NapkinAI.

## 4 Isolation of Biobased Polysaccharides with Microwaves

Biobased polysaccharides are complex macromolecules composed of long chains of monosaccharides linked by glycosidic bonds, which perform essential biological functions. Among their functions, their structural role stands out, as in the case of cellulose and hemicellulose, which give rigidity to cell walls, and energy storage, exemplified by starch.^52^ In addition, many natural polysaccharides have relevant bioactive properties, such as immunomodulation, antioxidant activity, antitumor activity, and prebiotic potential, making them objects of growing interest in the pharmaceutical, food, and cosmetic industries. Thus, the efficient and selective isolation of these polymers is essential to enable their technological applications, ensuring the purity, structural integrity, and functionality of the extracts obtained.^53^

The isolation of polysaccharides from natural matrices is traditionally carried out using conventional methods such as hot water extraction, organic solvent extraction, acid hydrolysis, and the use of specific enzymes. Although these processes are widely adopted, they have several practical limitations. Among the main challenges are long extraction times, which can take hours or even days, high energy and solvent consumption, and the potential compromise of polysaccharide quality due to thermal degradation and loss of volatile compounds.^15^ These characteristics make traditional methods less efficient and less sustainable, hindering industrial-scale production and increasing operating costs.

Microwave-assisted extraction (MAE) emerges as a promising and innovative alternative capable of overcoming these limitations. This technology uses high-frequency electromagnetic radiation to promote direct volumetric heating of the matrices. Unlike conventional heating, which depends on thermal conduction and convection from the surface to the interior of the sample, microwaves interact directly with the polar molecules and ions present, generating internal heat quickly and uniformly.^54^ This mechanism is based on dipole rotation and ionic conduction, which cause intense molecular movement and an increase in the temperature and pressure inside the cells. As a result, the cell walls are broken down, releasing the polysaccharides and facilitating their dissolution in the extraction medium.^49,55^

The results in the literature highlight the significant benefits of MNE. Ren et al.^56^ demonstrated that, using microwaves, extraction time can be reduced from 2 h to just 23 min, with an operating temperature reduced from 90 to 80 °C, thus minimizing the thermal degradation of compounds. This is especially important for heat-sensitive polysaccharides, whose chemical integrity and biological activity can be preserved through precise temperature control during the process. Chen and Xue^40^ reported a significant increase in yield of approximately 191%, as well as a drastic reduction in extraction time from 300 to 14 min, while maintaining biological properties such as antitumor activity, which reinforces the superiority of MSE over conventional methods.

Zhao et al.^19^ also highlighted that MAE allows the solvent/matrix ratio to be reduced from 1:40 to 1:30, optimizing resource use and increasing process sustainability, while extraction time was reduced from 5 h to just 6 min. Yield increased by about 46%, and microwave-extracted polysaccharides exhibited antibacterial activity greater than that obtained by traditional extraction. These findings indicate gains in not only efficiency but also functional quality of the isolated products.

The predominance of multimode microwave equipment in recent studies reflects the search for scalability and industrial viability, as these systems allow for the processing of larger volumes. However, adapted domestic microwaves are also frequently used in laboratories for preliminary optimization, provided that there is strict control of parameters such as temperature and pressure, which are fundamental to ensuring the reproducibility and safety of the process.^39,41,42^

A direct comparison between MAE and other modern techniques, such as ultrasound-assisted extraction, shows additional advantages. Hashemifesharaki et al.^41^ reported a 45.95% reduction in extraction time and a 19.42% increase in yield when using microwaves compared to ultrasound. Dong et al.^42^ confirmed these results, noting that microwave extraction reduces the time to one-third of that required for ultrasound extraction while increasing isolation efficiency.

Combinatorial approaches are also gaining prominence, with the combination of microwaves and ultrasound, as well as the introduction of adjuvants such as acids and surfactants in the extraction process. These combinations generate synergies that increase extraction efficiency, reducing the time and power required to achieve high yields. Zhang et al.^44^ and Yin et al.^57^ showed that the integration of techniques enables effective extractions even under milder conditions. Su et al.,^39^ for example, found a 17% increase in pectin extraction yield when applying hydrochloric acid in conjunction with microwaves, illustrating the potential of modulating the extraction medium to improve selectivity.

From a molecular point of view, polysaccharides have long and structurally rigid main chains, which limit their direct response to microwave energy. However, polar groups present in the side chains, such as hydroxyls, have sufficient mobility to absorb radiation through dipolar rotation, contributing to dielectric heating.^58^ In addition, the increase in temperature and the breaking of hydrogen bonds in the matrix facilitate the softening of tissues and increase the contact between the polymer and the solvent, promoting more efficient dissolution.^35^

Microwave-assisted extraction therefore not only provides significant gains in time and yield but also ensures the preservation of the biological activity of the extracted polysaccharides, a crucial factor for pharmaceutical and nutraceutical applications. The rapid increase in internal temperature and pressure induced by microwaves seems to favor the maintenance of the functional structure of polymers, despite occasional variations in molecular weight and conformation.^43,59,60^

A comparative overview of the extraction performance metrics reported for different extraction techniques is summarized in Table 3. In summary, microwave-assisted extraction represents a significant advance in the isolation of natural polysaccharides, combining efficiency, selectivity, functional preservation, and sustainability. Its application contributes to more economical and environmentally friendly processes, aligning with the current challenges of the biopharmaceutical and food industries in the production of high-quality, value-added natural biopolymers.

**Table 3:** Comparative Performance of Microwave-Assisted Extraction (MAE) and Conventional Extraction Methods for Biobased Polysaccharides

**Extraction method**	Conventional heating (reflux, hot water)	Ultrasound-assisted extraction (UAE)	Enzymatic extraction	Microwave-assisted extraction (MAE)
**Typical processing time (min)**	120–360	30–120	10–180	5–30
**Polysaccharide yield (%)**	5–20	10–30	15–35	20–45
**Energy demand**	High	Medium	Medium	Medium
**Main limitations**	Long extraction times, high energy consumption, possible thermal degradation	Limited penetration depth, efficiency dependent on particle size and viscosity	High enzyme cost, long processing times, sensitivity to pH and temperature	Risk of depolymerization under excessive power or prolonged irradiation
**Representative references**	^19^,^40,56^	^57^,^61^	^62–64^	^39^,^41^,^49^,^55^

## 5 Microwave-Assisted Chemical Modification of Polysaccharides

Microwave-assisted chemical modification of biobased polysaccharides is favored by the rapid and uniform transfer of energy directly to the reagents, reducing reaction times and minimizing thermal degradation of polysaccharides.^20^ In addition, microwave technology promotes better control of the reaction conditions, contributing to greater selectivity and reaction yields. In many cases, this method also eliminates or reduces the need for solvents, making the process more environmentally friendly.^21^ Among the main modifications performed are acetylation, phthalation, copolymerization, oxidation, esterification, sulfation, and quaternization reactions, as illustrated in Figure 4.

**Figure 4:**
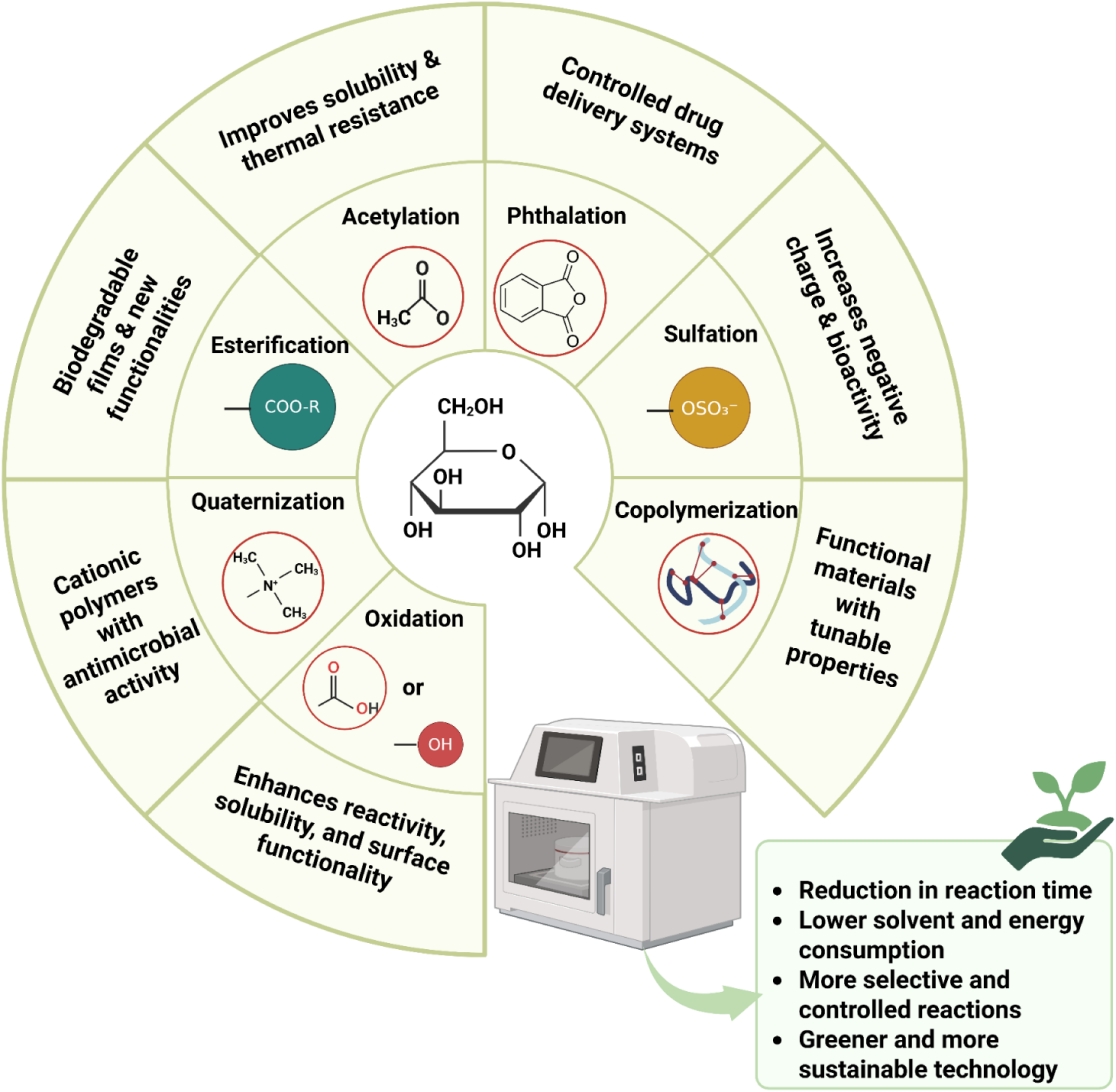
Chemical modifications using microwaves and their advantages.

Microwave heating uses electromagnetic radiation to rapidly heat water molecules and other polar substances present in the material. Unlike conventional methods, where heating is performed externally (by convection or conduction), microwaves act directly on the polysaccharide molecules, promoting more uniform and efficient heating.^65^ This process not only reduces reaction time but also allows chemical reactions to be carried out under milder conditions and with less degradation of polysaccharides.^66^

### 5.1 Acetylation

Acetylation is a chemical process in which acetyl groups (−COCH_3_) are added to the structure of polysaccharides through the esterification of their hydroxyls (O-acetylation) or to nitrogen atoms of monosaccharides with amine groups. The reaction aims to modify their physicochemical and functional properties, improving characteristics such as solubility, thermal resistance, hydrophobicity, and functionality, expanding the applications of these materials in various areas, mainly food, pharmaceuticals, and cosmetics.^67–70^

For the reaction to occur, polysaccharides must be solubilized in an aqueous or organic medium (such as formamide, dimethylformamide, and DMSO) and acetylated by the addition of acetylating agents, such as acetic anhydride. The process is favored by the addition of catalysts, making it faster and improving the degree of acetylation substitution.^71,72^ Several catalysts can be used in this process, among the most common are pyridine (which can also be used as a solvent), 4-dimethylaminopyridine (4-DMAP), and *N*-bromosuccinimide (NBS), the latter being notable for its lower cost and low toxicity.^73^ However, there is growing interest in sustainable and efficient alternatives for reaction catalysis, with a view to avoiding the production of toxic and polluting waste.^74^

Among the advantages of using microwaves in this context are energy savings, reduced processing time, and the possibility of carrying out reactions under milder conditions. Metal catalysts can be used in this context because they strongly absorb microwave radiation, heat quickly, and create a highly reactive environment, reducing the time and increasing the efficiency of the reaction.^75^ In this sense, it is observed that the degree of polysaccharide substitution is directly proportional to the concentration of the metal used for catalysis.^76^ Furthermore, in systems where liquid catalysts, such as weak acids or bases, are used, microwaves can intensify the ionization of these species, increasing reactivity without the need for stronger catalysts or larger quantities.^77^

Applications of acylated polysaccharides include areas such as food, cosmetics, pharmaceuticals, biodegradable packaging, and materials engineering. For example, acylation of cellulose can result in cellulose esters used as coatings or filter membranes, while acylated chitosan is applied in controlled drug delivery systems due to its biocompatible and biodegradable properties.

### 5.2 Phthalation

The introduction of phthalic groups (derived from phthalic acid or its anhydrides, such as phthalic anhydride) into the structure of biopolymers is mainly used to modify solubility and the potential for interaction with other substances and receptors. This modification enhances the use of biopolymers in the composition of targeted drug delivery systems.^78^ In addition, the improvement of thermal properties and plasticity and flexibility are possible applications resulting from the introduction of phthalic groups into their structure.^79^

The reaction occurs through the interaction of phthalic anhydride with nucleophilic groups present in the biopolymer (hydroxyl, amine, or carboxyl) under suitable temperature and solvent conditions. This results in the formation of an ester bond (with −OH groups) or an amide bond (with −NH_2_ groups), incorporating the phthalic group into the biopolymer. Acids (such as H_2_SO_4_) or bases (such as pyridine) can be used to facilitate the reaction. In addition, organic solvents such as DMF (dimethylformamide) or acetone are commonly used to dissolve the reagents and promote the reaction.^80^

The use of microwaves allows the reaction to start at lower temperatures, enabling high performance more quickly and efficiently than with the conventional method. In addition, adjusting parameters such as power and time can help control the degree of substitution of the product obtained.^81^

### 5.3 Copolymerization

Copolymerization is a chemical process in which two or more different types of monomers are combined to form a single polymer chain. This reaction allows the creation of materials with adjustable and specific properties that would not be obtained with a polymer composed of only one type of monomer (homopolymer).^22^ When applied to biopolymers, this approach can lead to the creation of materials with improved properties, such as greater strength, biocompatibility, or functionality for specific applications.

Copolymerization by free radicals is a widely used process that utilizes free radicals as reactive intermediates to promote the reaction between two or more monomers. The reaction begins with the generation of free radicals, usually by the decomposition of initiators such as peroxides or azo compounds, by thermal, photochemical, or redox methods.^82^ These radicals attack the double bonds of the monomers, forming active radicals that promote the growth of the polymer chain. During propagation, monomers are successively added to the growing chain, and the distribution of monomers depends on the cross-reaction rates, which are defined by the reactivity parameters of each monomer. The process ends when free radicals are eliminated by recombination or disproportionation, interrupting the chain growth. Free radical copolymerization allows different types of copolymers to be obtained, such as random, alternating, block, or graft copolymers, depending on the reaction conditions and the nature of the monomers.^83^

The use of initiators in copolymerization reactions presents challenges related to their efficiency and control. Many initiators require specific conditions, such as high temperatures, radiation, or specific solvents, which can increase the costs and complexity of the process. In addition, their decomposition can generate unwanted byproducts, compromising the purity of the final copolymer. Another problem is the control of the reaction rate and molecular weight of the polymer, which can be affected by variations in the concentration or reactivity of the initiator, making it difficult to obtain materials with consistent properties.^66^

The copolymerization of biopolymers using microwaves follows the same basic principle as conventional polymerization reactions, but the method can also help overcome problems associated with initiators by promoting their decomposition more efficiently, without the need for extremely high temperatures.^84^ This reduces reaction time, minimizes the formation of unwanted byproducts, and improves control over the composition and molecular weight of the copolymer.^85^

As with other types of reactions, the chemical structure and physicochemical properties of the reactants can favor the performance of the reaction. Regarding the chemical structure of the monomer to be used, organometallic monomers are more influenced by electromagnetic radiation, which significantly increases the reaction rate and causes differences in the composition of the resulting copolymer.^86^ As for the solvent used, its dielectric constant has a major impact on the reaction yield. Solvents with a high dielectric constant, such as dimethylformamide (DMF), interact strongly with microwave radiation, heating up quickly. Thus, by selecting the appropriate solvent for microwave-assisted polymerization, it is possible to optimize the method, reducing the energy required without significantly impacting the properties of the polymer.^87^

### 5.4 Esterification

As a simple alcohol, the hydroxyl groups of polysaccharides can be esterified in reactions with fatty acids or acid derivatives (such as anhydrides), allowing the formation of esters. This process improves the solubility and interaction of the polysaccharide with other compounds, making it useful for applications in biodegradable films and other materials.^88,89^

The esterification reaction is based on the formation of covalent bonds between the hydroxyl groups of the polysaccharide and carboxylic compounds, such as organic acids or their derivatives, such as anhydrides or acid chlorides. The mechanism involves activation of the carboxyl group to facilitate its reaction with the hydroxyls available in the polysaccharide. The most common reagents include organic acids (e.g., acetic acid or citric acid), anhydrides (such as acetic anhydride), and acid chlorides (such as acetyl chloride).^90^

The type of reagent chosen depends on the desired degree of functionalization and the specific properties to be incorporated into the material. The use of solvents is also critical to ensuring homogeneous dispersion of the reagents and reaction efficiency. Solvents such as dimethyl sulfoxide (DMSO), acetone, methanol, and water are often used, depending on the solubility of the polysaccharide and the reactivity of the system.^89^

During the process, it is common to use catalysts that accelerate the reaction and increase the degree of substitution in addition to improving selectivity. In this sense, mineral acids, such as sulfuric acid or hydrochloric acid, and organic catalysts, such as pyridine, can be used. Enzymatic catalysts, such as lipases, have also been explored in green esterifications, offering greater selectivity and milder reaction conditions.^91,92^

Control of reaction conditions, such as the temperature, time, and molar ratio between reagents, is essential to obtain a final product with the desired characteristics. Polysaccharide esterification is a versatile tool that allows the creation of functionalized materials for specific applications, standing out for its ability to modify key properties of biopolymers in an effective and customizable way.^93^

This type of reaction can also be exploited in the reuse of industrial waste. In this regard, studies^94^ compared the results of the esterification process of cotton cellulose waste with the aim of obtaining biodegradable plastics. The process used *N*,*N*-dimethylacetamide/lithium chloride (DMAc/LiCl) as a solvent and *N*,*N*-dimethyl 1-4-aminopyridine as a catalyst. Considering the energy and time savings, the satisfactory product yield, and the substitution degree values, the use of microwaves proved to be an efficient heating source to facilitate cellulose esterification, reducing the reaction time from 2 to 12 h to just 3 min.

Lukasiewicz and Kowalski^95^ associated the use of enzymes with microwaves for the esterification of starch with acetic, lauric, or stearic acid in the presence of lipase. Lipases are enzymes widely used in esterification, ester hydrolysis, or transesterification processes, which have interesting thermal stability and high activity, even when organic solvents are used as the reaction medium. Low-power microwave irradiation (80–160 mW/g of sample) with the solvent DMF resulted in a higher degree of substitution and less degradation of the polysaccharide.

### 5.5 Oxidation

The oxidation of polysaccharides is a chemical modification widely used to alter their physicochemical properties, such as solubility, surface charge, reactivity, and functionality. This process involves the introduction of oxygenated functional groups, such as aldehydes, ketones, or carboxyls, into the structure of polysaccharides, partially replacing the hydroxyl groups. Oxidation is essential for producing polysaccharide derivatives with applications in areas such as biomedicine, food, cosmetics, and water treatment.^61,96^

The basic principle of the oxidation reaction lies in the controlled removal of electrons from specific carbon atoms in the polysaccharide, usually in glucose units or their derivatives. Depending on the type of oxidizing agent used, the reaction can occur at different sites in the structure. For example, oxidation with sodium periodate (NaIO_4_) results in the cleavage of vicinal C–C bonds, generating dialdehyde groups, while oxidation with sodium hypochlorite (NaClO) in the presence of specific catalysts, such as 2,2,6,6-tetramethylpiperidine-1-oxyl (TEMPO), converts primary hydroxyl groups into carboxyl groups.^97,98^

The most common oxidizing reagents in polysaccharide modification include sodium hypochlorite (NaClO), widely used in the oxidation of materials such as cellulose and chitosan, especially in TEMPO-catalyzed systems, due to its efficiency and selectivity.^99^ Sodium periodate (NaIO_4_) is another frequently used oxidizing agent known for its ability to perform selective oxidations that generate dialdehyde groups in the polysaccharide structure. Hydrogen peroxide (H_2_O_2_), on the other hand, is an environmentally friendly and efficient oxidant, applied under mild conditions for gentle and sustainable modifications. Solvents vary depending on the solubility of the polysaccharide and the oxidant. Aqueous systems are the most common due to their environmental and economic compatibility, but organic solvents, such as dimethyl sulfoxide (DMSO) and acetone, are also used in specific cases.^100^

Controlling reaction conditions such as pH, temperature, and time is essential to prevent excessive degradation of the polysaccharide and ensure that products with optimized properties are obtained.^101^ Polysaccharide oxidation is a versatile strategy for producing advanced functional materials with applications in different industries. In this regard, Lin et al.^102^ successfully obtained TEMPO-oxidized cellulose granules with adsorption capacity for organic dyes, reducing the reaction time from 36 h using the traditional method to just 6 h using a microwave reaction.

In addition to reducing reaction time, microwave initiation can reduce the need for solvents and catalysts, as noted by Komulainen et al.^103^ In this case, microwave activation was shown to significantly increase the oxidizing power of bromate, drastically reducing the amount of bromate required to only one-tenth of the oxidant used at room temperature when the reaction was conducted at 60 °C. In addition, the reaction time was shortened from 2 to 5.5 h to just 10 min, resulting in comparable amounts of efficiently oxidized products.

### 5.6 Sulfation

The process of polysaccharide sulfation occurs through the introduction of sulfates into the hydroxyl groups available in their structure. This modification increases the negative charge of polysaccharides, which can improve their solubility in water and their interaction with proteins, enzymes, and other biological compounds. Among the main applications of sulfated polysaccharides, their biological applications stand out, such as their use in anticoagulants and in the study of antitumor, antioxidant, and immunomodulatory agents.^104^

The main sulfating reagents include sulfuric anhydride (SO_3_), chlorosulfuric acid (ClSO_3_H), and sulfuric acid (H_2_SO_4_). In some approaches, ammonium sulfate can also be used, which, in combination with acids, provides sulfate ions for the modification. For reactions requiring anhydrous (water-free) conditions, organic solvents such as dimethylformamide (DMF) or pyridine are used, which also help to dissolve the reagents and polysaccharide.^105^

Pyridine is a commonly used catalyst for this reaction because, in addition to activating the sulfating reagents, it also neutralizes the acidic byproducts that may arise during the reaction. In some approaches, sulfuric acid acts as both a reagent and a catalyst. Reaction conditions, such as the temperature and time, are adjusted to preserve the polysaccharide structure and control the degree of sulfation. Typically, the temperature range is from 30 to 100 °C, while reaction time can range from minutes to several hours, depending on the reactivity of the reagents and the thermal resistance of the material. This control is essential to ensure that the final product maintains its structural and biological properties, in addition to presenting the degree of sulfation necessary for its desired application.^106,107^

As with other chemical modifications, the use of microwaves stands out in relation to conventional methods, especially due to its greater efficiency and lesser impact on the structure of the material. In addition to the advantages already presented, a study conducted by Xing et al.^108^ observed that microwave radiation preserves the integrity of the pyranose rings in polysaccharides, preventing structural degradation that can be caused by traditional methods. Furthermore, microwaves do not alter the type of chemical groups substituted in chitosan, ensuring that the modification occurs in a selective and controlled manner, similar to a conventional process. These factors make the microwave-assisted process a more efficient, sustainable, and reliable approach for the chemical modification of polysaccharides.^108^

### 5.7 Quaternization

Quaternization consists of replacing the hydroxyl groups (−OH) present in monosaccharide units with a quaternary ammonium reagent. This group contains a positively charged nitrogen, which forms a total of four bonds.^109^ The main applications of this type of modification are to increase the solubility of polysaccharides in water and to produce materials with antimicrobial properties. The latter application results from the interaction of the positive charge of the quaternary groups with the negative charge of the cell membrane of microorganisms, destabilizing it.^110,111^

To incorporate ionic groups into polysaccharides, cationic reagents containing functional groups such as imino, ammonium, phosphonium, sulfonium, or amino are commonly used. Among these, (3-chloro-2-hydroxypropyl)­trimethylammonium chloride (CHPTAC) stands out as one of the most widely used agents in the synthesis of cationic polysaccharides due to its low cost, low toxicity, and good stability.^112^

In this process, CHPTAC is initially converted to 2,3-epoxypropyltrimethylammonium chloride (EPTAC) before the quaternization reaction. This conversion is usually performed using sodium hydroxide (NaOH), which facilitates the closure of the chlorohydrin ring present in CHPTAC. However, an inadequate molar ratio between NaOH and CHPTAC can compromise the efficiency of this conversion process, because high concentrations of NaOH can trigger secondary reactions with CHPTAC, forming byproducts due to epoxide hydrolysis, while high concentrations of CHPTAC reduce the degree of substitution of the reaction, since the limited amount of NaOH prevents the complete conversion of CHPTAC to epoxide.^113^

With regard to solvents, aqueous systems are commonly used due to the solubility of the reagents and environmental compatibility, but organic solvents such as isopropanol can also be used to improve the dispersion of polysaccharides and promote the reaction.^114^ In addition, other catalysts, such as alkalis and acids, can be used in specific formulations depending on the characteristics of the polysaccharide and the desired degree of substitution. These components play crucial roles in controlling the reactivity, selectivity, and efficiency of the quaternization process.^115^

Due to indirect heating and low heat transfer efficiency, the quaternization reaction of polysaccharides performed by conventional methods can take several hours, often between 4 and 24 h, depending on reaction conditions such as temperature, reagent proportions, and type of solvent used.^116^ The use of microwaves allows for uniform and direct heating of the medium, increasing the speed of chemical reactions and improving control of reaction conditions, reducing the time required to minutes, usually less than 30 min, without compromising the yield or quality of the final product.

The production of water-soluble chitosan derivatives using microwave heating is particularly noteworthy in this regard. The production of *N*-(2-hydroxy)-propyl-3-trimethylammonium chitosan chloride (QCh) using epoxypropyl trimethylammonium chloride (EPTMAC) in isopropyl alcohol was carried out in just 50 min under microwave irradiation, allowing the production of a structurally similar product to that obtained by the conventional heating method in a 6-h reaction.^114^ Another study used glycidyl trimethylammonium chloride (GTMAC) in an acidic medium to obtain the same product, in this case reducing the time to approximately 30 min. In addition, spectroscopic analysis of QCh confirmed that no additional chemical modification occurred as a result of the reaction between chitosan and GTMAC, preserving the structural integrity of the material.^117^

## 6 Advantages, Limitations, and Challenges of the Technique

The use of microwaves in the isolation and modification of biobased polysaccharides offers several advantages but also faces some challenges that need to be overcome for its widespread application in laboratories and industries. Among the main advantages is energy efficiency, since microwave heating is direct and selective, significantly reducing energy consumption compared to conventional methods.^118^ In addition, the reaction time is considerably shorter, allowing processes that would traditionally take hours to be completed in minutes. The technique also provides greater control of reactions, ensuring thermal uniformity and minimizing the degradation of polysaccharides.^116^

The use of microwaves for the isolation and chemical modification of polysaccharides is consistent with several principles of green chemistry. First, it is related to energy efficiency, as microwave radiation allows for rapid and selective heating of materials, significantly reducing energy consumption compared to conventional methods.^23^ It also complies with the principle of prevention by minimizing the formation of unwanted waste and byproducts due to precise control of reaction conditions.^24^ The technology also promotes the use of safer solvents, as many microwave-assisted reactions can be carried out in water or green solvents, replacing toxic solvents. In addition, the principle of using catalysts is favored, as the technique improves the efficiency of catalysts, reducing the amount needed.^71^ Finally, the process is consistent with the reduction of unnecessary steps, since the acceleration of the reaction eliminates the need for long and auxiliary steps, making the methods simpler and more sustainable. These aspects make the use of microwaves a practical and environmentally friendly approach to the treatment of polysaccharides.

The technique becomes even more advantageous when combined with the use of green solvents, so-called because they are designed to minimize environmental impacts by reducing toxicity, improving safety, and promoting sustainability in chemical processes.^119^ They are part of the principles of green chemistry, seeking to replace conventional solvents that are often toxic and polluting. They are characterized by being biodegradable, renewable, nonvolatile, and less hazardous to the environment and human health.^120^ Common examples include water, ethanol, eco-friendly ionic liquids, and deep eutectic solvents (DES).

Water is the most abundant and nontoxic solvent, ideal for chemical reactions and extractions.^121^ Microwave irradiation is becoming a viable method for extracting polysaccharides using only compressed hot water.^122^ Ethanol, obtained from renewable sources, is biodegradable, has low toxicity, and is effective in various organic reactions, especially in microwave-assisted systems due to its high dielectric constant.^123^

Eco-friendly ionic liquids, composed of cations and anions with low melting points, are designed to be nonvolatile and recyclable, making them a safe alternative to conventional organic solvents.^124^ DES, formed by eutectic mixtures of natural compounds, offer high biodegradability, low cost, and ease of preparation, making them ideal for biopolymer extractions and modifications.^125,126^ In this sense, these solvents also stand out for their excellent performance in solubilizing polysaccharides such as cellulose, which are considerably difficult to solubilize even in organic solvents.^127^

When it comes to their use in microwaves, polar green solvents, such as water, ethanol, and ionic liquids, heat up quickly through dipole rotation, while solvents containing free ions, such as ionic liquids and some deep eutectic solvents (DES), heat up mainly through ionic conduction. This ability to interact efficiently with microwaves makes green solvents an ideal choice for green chemistry processes, combining sustainability with energy efficiency.^128^ Thus, their use not only contributes to the reduction of waste and emissions but also makes processes more economical and aligned with the principles of sustainability.

On the other hand, the technique presents challenges that need to be considered. The initial cost of specialized equipment can be high, hindering its adoption on a small scale, while the transposition of laboratory processes to an industrial scale also requires technical and economic adjustments.^129^ From an industrial perspective, the implementation of microwave-assisted technologies has significant implications in terms of both capital expenditure (CAPEX) and operational expenditure (OPEX). Although industrial microwave reactors generally require higher initial CAPEX compared to conventional heating systems, numerous studies report substantial reductions in processing time, often on the order of 60–90%, which directly translates into increased volumetric productivity.^130,131^ In addition, shorter extraction times combined with the higher energy efficiency of volumetric microwave heating contribute to significant reductions in energy consumption, positively impacting OPEX. The ability to operate with lower solvent volumes and shorter times further decreases costs associated with solvent recovery and effluent treatment, as observed by Wu et al.^126^ and Ajami et al.^132^ Taken together, these factors suggest that despite higher initial investment, microwave-assisted processes can offer competitive payback times and strong economic viability for large-scale industrial applications.

Despite the advantages associated with microwave-assisted extraction, this technology presents inherent technical limitations that must be carefully considered during process design and optimization. In larger samples, uniform heating can be a problem, compromising reaction control.^133^ In addition, not all reagents or substrates absorb microwaves efficiently, which limits their use in certain cases. Operational safety is also an important factor, as rapid reactions can generate high temperatures and pressures, requiring appropriate equipment to ensure safety.^134^

Another critical point is the need for careful optimization of reaction conditions, such as power, time, and system composition, mainly to avoid degradation of components in the system and reduce the production of byproducts.^135^ The application of excessively high microwave power or prolonged irradiation times can induce significant polysaccharide degradation, primarily manifested as a reduction in average molecular weight and loss of bioactivity, particularly for thermolabile polysaccharides.^136^ In multimode microwave systems, additional challenges include the formation of temperature gradients and localized hotspots, which may compromise heating homogeneity and process reproducibility at larger scales. In this context, molecular weight preservation (Mw) emerges as a critical quantitative performance metric for future advances in the field, as it directly reflects the balance between extraction efficiency and structural integrity.^137^ Precise control of operational parameters, combined with systematic structural characterization, is therefore essential to mitigate these limitations and enhance the robustness and industrial applicability of microwave-assisted extraction.

Despite this, the use of microwaves has great potential and, with technological advances and greater investment in research, could become an indispensable tool in sustainable chemistry and in various industrial applications.^138^ In addition, the ability to precisely control the temperature during the isolation and modification process allows for the optimization of reaction conditions, resulting in higher quality products.^16^ Microwave-assisted chemical modification of natural polysaccharides represents a promising approach that not only optimizes industrial processes but also contributes to sustainability and the reduction of environmental impacts.^139^ This technology has the potential to transform the way polysaccharides are isolated, modified, and applied in various industries, promoting innovation and the creation of new products with better functional and environmental characteristics.

## 7 Environmental and Economic Aspects, Trends, and Future Outlook

The use of microwaves in the isolation and modification of biobased polysaccharides represents a promising trend in the search for more sustainable, efficient, and versatile processes in biopolymer chemistry, becoming a competitive alternative to traditional methods.^108^ Future trends and prospects involve advances in areas such as solid-phase reactions, the use of green solvents, and solvent-free reactions, each playing a role in process optimization.^122^

Solid-phase reactions, where the reactants are in the solid state, eliminate or significantly reduce the use of solvents.^140^ This is highly desirable from an environmental and economic standpoint. Microwave radiation is particularly effective in this context, as it directly heats solid materials evenly, accelerating reactions that traditionally require long times or high temperatures.^141^

Another interesting aspect of microwave use includes the use of green solvents, which, when combined with microwave-assisted heating, improve reaction efficiency and reduce the generation of hazardous waste.^128^ Solvent-free reactions are highly desirable in terms of sustainability, eliminating the need for solvent recovery and disposal.^21^

The main advantages of solvent-free microwave reactions include accelerated reaction rates due to high heating rates, resulting in significantly reduced byproducts and higher reaction yields. In addition, reaction conditions are versatile, allowing for both low-power and low-temperature synthesis (mild conditions) and high temperatures and pressures (autoclave conditions). The distinct heating abilities of microwaves generate different reaction selectivities, and the quick and easy handling of experimental parameters provides excellent reproducibility.^142^ The conjugation of fatty acids to pectin without solvents has already been reported in the literature as a promising method for modifying polysaccharides, reducing costs and environmental impact.^138^

Among the main future trends in the use of this technique are the development of hybrid processes, combining the use of microwaves with ultrasound, high pressures, or enzymatic catalysts to improve the yield and selectivity of reactions with polysaccharides.^62^ The scalability of this process also deserves attention, as advances in the design of industrial-scale microwave reactors are enabling the transition of these processes from laboratories to large-scale applications.^143^

The integration of microwave use with biorefinery approaches to maximize the use of agro-industrial waste in the production of polysaccharides also stands out as a future prospect.^144^ In this sense, the economic viability of the microwave-based biorefinery process has shown that despite the initial costs involved in installing the machinery, the integrated biorefinery process offers remarkable technical, environmental, and economic advantages, making it profitable from an industrial perspective. Thus, upscaling is not only technically feasible but also financially profitable, regardless of whether multiple products or a single product are produced.^129^

The use of microwaves in solid-phase reactions, with green solvents or without solvents, aligns with the principles of green chemistry, promoting energy efficiency, reducing the associated carbon footprint, minimizing waste reduction, and decreasing the use of toxic substances.^131^ These approaches represent the future of biopolymer modification, contributing to the development of innovative and environmentally friendly materials for advanced applications such as nanomaterials, biomaterials, and adsorbents for environmental remediation.^13^

## 8 Conclusion

The isolation and chemical modification of natural polysaccharides is a constantly evolving field, with applications ranging from the food industry to pharmaceuticals, as well as playing a crucial role in new biomaterials and sustainable technologies. These biopolymers, which are widely abundant in nature and easily accessible, have proven to be promising resources due to their biodegradability, functional versatility, and structural properties. Advances in chemical modification technologies, such as the use of microwaves, have emerged as an innovative solution to optimize the extraction and modification processes of these materials, standing out for their advantages in energy efficiency, reduced reaction time, and greater control over the reaction parameters.

Microwave technology stands out in the context of polysaccharide modification because it allows direct and highly efficient heating of systems, resulting in faster and more reproducible reactions. This feature makes processes more economical and environmentally friendly, especially when combined with green chemistry approaches such as the use of green solvents or even solvent-free reactions. This versatility enables the creation of biopolymers with adjusted properties, such as greater thermal stability, mechanical resistance, and specific functional properties, which are applicable to a wide range of industries, from medicines and biodegradable adhesives to advanced materials.

In addition, the application of microwaves allows the exploration of new avenues for the chemical modification of polysaccharides, aiming to improve their rheological properties, such as viscosity, or to provide bioactive characteristics, such as antioxidant and antimicrobial activity. These modifications are often necessary to adapt polysaccharides to new industrial applications, for example, in biodegradable films and coatings, which meet the growing demand for sustainable materials.

The use of microwaves has also proven to be effective in solid-phase reactions, an advantageous approach when seeking to avoid excessive use of organic solvents or when wishing to promote specific reactions in solid compounds. Microwave radiation directly heats the compounds, accelerating reactions and increasing the overall efficiency of the process. This method not only reduces costs and environmental impacts but also improves the productivity and purity of the final products, generating interest in its application in greener and more sustainable industrial processes.

However, despite its potential, the use of microwaves for modifying biobased polysaccharides still presents significant challenges. The initial cost of microwave equipment, the scalability of the technology for large volumes, and the homogeneous distribution of microwave radiation in larger systems are issues that need to be addressed for these processes to be adopted on a large scale. The compatibility between natural polysaccharides and microwave methods also needs to be further explored. Although most of the materials present in the reactions are good microwave absorbers, the efficiency of the technique depends on the structure and composition of the polysaccharide, as well as the type of reaction desired.

On the other hand, investments in research and development in the field of microwaves have driven a series of advances. The integration of microwaves with other technologies such as ultrasound, high pressures, or specific catalysts has shown promising results in terms of increased reactivity and reaction control. In addition, the development of new reaction protocols and process standardization are fundamental steps toward optimizing the industrial application of microwave technology in natural polysaccharide modification systems.

In terms of future prospects, the potential of microwaves in modifying biobased polysaccharides can be further explored as new control methodologies are developed. The use of microwaves can, for example, be combined with biocatalysis techniques, resulting in more specific processes with less environmental impact. The incorporation of microwaves into biorefinery processes also presents itself as a promising area where the conversion of natural biomass into higher-value-added products, such as bioplastics, food additives, and pharmaceuticals, can be accelerated and made more efficient.

In addition, the growing interest in sustainable solutions and the search for alternatives to traditional plastics and other polluting materials reinforce the importance of research on modified polysaccharides. These materials, increasingly sought after for their biodegradable and renewable properties, are positioned as protagonists in various industrial innovations, and microwave-assisted modification offers a promising path to meet this increasing demand.

The application of microwaves in the isolation and chemical modification of biobased polysaccharides represents a revolution in the field of polymer chemistry, bringing remarkable benefits in terms of efficiency, sustainability, and versatility. Although there are challenges to be faced, continuous advances in this area offer great potential for creating new sustainable materials, contributing to the transition to a greener and more circular economy. The combination of microwaves with green chemistry approaches and the exploration of new reagents and catalysts are trends that can further expand the frontiers of the use of modified natural polysaccharides, making them essential components in various industries in the future.
